# Botanical from the medicinal spice, *Piper capense* is safe as demonstrated by oral acute and subchronic toxicity investigations

**DOI:** 10.1016/j.heliyon.2020.e05470

**Published:** 2020-11-09

**Authors:** Brice E.N. Wamba, Armelle T. Mbaveng, Ghislain M. Tazoho, Victor Kuete

**Affiliations:** Department of Biochemistry, Faculty of Science, University of Dschang, Cameroon

**Keywords:** *Piper capense*, Toxicity, Acute, Sub-acute, LD50, Biochemical parameters, Alternative medicine, Biological sciences, Cancer research, Diet, Evidence-based medicine, Health sciences, Hematological system, Toxicology

## Abstract

*Piper capense* Linn is a plant used in Cameroon to treat cancer and several other diseases such as urinary tract disorder, fever, stomach-ache and to improve appetite. The methanol extract of *Piper capense* has been reported for its antiproliferative activity towards several human cancer cell lines. The aim of this work was to evaluate the acute and subchronic oral toxicities of a methanol extract from *P. capense* fruits on rats. The acute oral toxicity assay was carried out by administration of a single dose of 5000 mg/kg body weight of methanol extract of the *Piper capense* to five female rats, after which the behavior of the animals and the number of deaths were noted after 48 h. The animals were then kept for observation for 14 days. On the 15^th^ day, the rats were sacrificed and macroscopic observation of the organs was made. Concerning the subchronic toxicity study, the rats composed of males and females received three doses (250, 500 and 1000 mg/kg body weight/day) for a period of 28 days by oral gavage. General animal behavior, food intake, weight gain, organ weights, haematological parameters, serum, and urinary biochemical parameters, and histological sections of liver and kidneys, were evaluated. Methanol extract from the *Piper capense* fruits did not cause any death in rats that were administered a single dose of 5000 mg/kg body weight of extract and therefore, the letal dose 50 (LD50) of the extract is greater than 5000 mg/kg body weight. Subchronic administration of the methanol extract of *Piper capense* fruits showed significant variations (P > 0.05) after analysis of certain biochemical parameters: serum urea, urinary urea, alanine aminotransferase (ALAT), aspartate aminotransferase, (ASAT), serum protein; in both male and female rats that received the dose of 1000 mg/kg body weight/day. No major signs of toxicity were observed in the liver and kidneys of animals after analysis of the histological sections performed. Beside, some signs of toxicity were observed, including cell lysis and inflammation on the liver and kidney organs at a dose of 1000 mg/kg body weight/day. Finally, the methanol extract of *Piper capense* fruits is safe at lower doses, but could cause some damages at doses as high as 1000 mg/kg body weight/day. Consequently, it should be taken with caution when used in therapy.

## 1 Introduction

Secondary metabolites contained in plants due to their multiple biological activities are increasingly the preferred target in the scientific community's search for new and effective drug substances (Cowan, 1999). Several scientific studies have demonstrated the pharmacological properties of a number of medicinal and edible plants (Wamba et al., 2018; Bobasa et al., 2018; Mbaveng et al., 2018). However, only a few studies published have investigated the *in vivo* toxicity of food spices. Food spices are used to treat diseases such as diabetes, cancer, and bacterial infections (Kuete et al., 2013; Fankam et al., 2011).

*Piper capense* L.f. (Piperaceae) is used in many countries to treat various ailments such as cancer, urinary disorder, fever, stomach-ache and to improve appetite (Eyob et al., 2018; Mbaveng et al., 2017; Kuete and Efferth, 2015; Woguem et al., 2013; Kuete et al., 2011, 2013). A methanol extract of the fruits has been reported to have a cytotoxic effect towards a panel of human cancer cell lines, including multidrug resistant phenotypes (Kuete et al., 2011, 2013). These cell lines included CCRF-CEM (IC_50_: 6.95 μg/mL), HL60 (IC_50_: 8.16 μg/mL), HL60AR (IC_50_: 11.22 μg/mL) and CEM/ADR5000 (IC_50_: 6.56 μg/mL) leukemia cell lines, MDA-MB231 (IC_50_: 4.17 μg/mL) and MDA-MB231/*BCRP* (IC_50_: 19.45 μg/mL) breast adenocarcinoma cell line, HCT116 *p53*^+/+^ (IC_50_: 4.64 μg/mL) and HCT116 *p53*^−/−^ (IC_50_: 4.62 μg/mL) colon carcinoma cell line, U87MG (IC_50_: 13.48 μg/mL) and U87MG.*ΔEGFR* (IC_50_: 7.44 μg/mL) gliobastoma cell line, Hep-G2 (IC_50_: 16.07 μg/mL) hepatocarcinoma cell line and Mia Paca2 (IC_50_: 8.92 μg/mL) pancreatic cancer cell line (Kuete et al., 2011, 2013). In addition, the oil from the fruits of *Piper capense* displayed antiproliferative effects towards MDA-MB 231 cells (IC_50_: 26.3 μg/mL), A375 melanoma cells (IC_50_: 76.0 μg/mL) and HCT116 cells (IC_50_: 22.7 μg/mL) (Woguem et al., 2013). The methanol extract of the bark displayed antibacterial activity against the Gram-positive cocci, *Staphylococcus aureus,* and *Staphylococcus epidermidis,* with a minimal inhibitory concentration (MIC) of 0.52 mg/mL (Steenkamp et al., 2007). The methanolic extract of the fruits showed activity against a panel of Gram-negative bacteria including sensitive and resistant phenotypes such as: *Escherichia coli* ATCC10536 (MIC: 256 μg/mL), *E. coli* AG100, AG100A_tet_, AG102, MCC4100 and W3110, *Enterobacter aerogenes* EA3 and EA27 (MIC: 1024 μg/mL), *E. aerogenes* EA298 (MIC: 512 μg/mL), *E. aerogenes* EA294 (MIC: 256 μg/mL), *Enterobacter cloacae* BM47 and BM67 (MIC: 512 μg/mL), *Klebsiella pneumoniae* ATCC11296, KP55 and K24 (MIC: 1024 μg/mL), *K. pneumoniae* K2 (MIC: 512 μg/mL), *K. pneumoniae* KP63 (MIC: 256 μg/mL), *Providencia stuartii* ATCC29914 (MIC: 1024 μg/mL), and *P. stuartii* NAE16 (MIC: 256 μg/mL) (Fankam et al., 2011). The methanol extract of the aerial parts of the plant displayed antiplasmodial activity against *Plasmodium falciparum* chloroquine-resistant strain (W2) with an IC_50_ value of 7 μg/mL (Kaou et al., 2008). The methanol extract of *Piper capense* fruits (or roots) had 2,2-diphenyl-1-picryl hydrazyl (IC_50_ value of 0.040 mg/mL) and 2,2′-azinobis-3-ethylbenzothiazoline-6-sulfonic acid (IC_50_: 0.044 mg/mL) radical scavenging activity while an ethyl acetate extract had acetylcholinesterase inhibitory activity with an IC_50_ value of 0.041 mg/mL (Kilkenny et al., 2010). In addition to the multiple biological activities that a plant can possess, the toxicological study of the plant is important because it provides information about the dose that can cause damage in a living organism.

Despite this impressive antiproliferative and antibacterial potentials of *Piper capense*, no scientific study has focused on its possible harmful effects. Hence the objective of this study was to evaluate the acute and subchronic oral toxicities of a methanol extract of the fruits of *Piper capense* on *Wistar* strain rats.

## 2 Material and methods

### 2.1 Collection and identification of plant material

The fruits of *Piper capense* used in this work were purchased at the Dschang local market (5°27′N 10°04′E) in April 2018 (Menoua division of the West region of Cameroon). The plant was subsequently identified and authenticated at the National Herbarium of Cameroon (NHC), Yaoundé, where a sample was deposited and registered under reference number 6018/SRF-Cam.

### 2.2 Preparation of the plant extract

The plant was ground and the powder obtained macerated in methanol in the proportions 1:3 (w/v) for 48 h at room temperature followed by filtration using Whatman No. 1 paper. The filtrate obtained was evaporated using a rotary evaporator (BÜCHI R-200) at 65 °C allowing recovery of a crude extract that was stored at -25 °C; after evaporation of the solvent in the rotary evaporator, the extract was dried in an oven for 4–6 h to allow complete vaporization of the residual solvent.

### 2.3 Experimental animals

Animals were obtained from the Animal Breeding Laboratory, Faculty of Science, University of Dschang. Animals were housed individually in polypropylene cages with soft bedding in the housing facility. Five (5) female albino *Wistar* strain rats aged between 8 and 12 weeks with an average weight of 170 g were used for the acute oral toxicity study. Concerning the subchronic oral toxicity study, a total of forty (40) albino rats of both sexes (20 males and 20 females) of *Wistar* strain aged between 8 and 12 weeks, with an average weight of 170 g were used. They were maintained in an animal room at temperature of 20 ± 2 °C under a standard animal room condition of 12 h light/dark cycle; the animals were raised in the animal house of the Biochemistry Department of the University of Dschang. They were fed with a standard rat feed and had free access to water. The acclimatization of the animals lasted two weeks, during which time it was ensured that they were in good health before testing. There is no ethical committee regarding research on animals in Cameroon. The study was conducted according to the guidelines of the European Union Institutional Ethics Committee on Animal Care (Council EEC 86/609). All sections of this report adhere to the ARRIVE Guidelines for reporting animal research (Adewusi, and Steenkamp, 2011) (Additional file 1). A completed ARRIVE guidelines checklist is included in Checklist S1.

### 2.4 Acute oral toxicity study

The acute oral toxicity of the methanol extract of *Piper capense* fruits was performed according to the protocol described by Organization for Economic Cooperation and Development (OECD) 2008, Guideline 425 Limiting Test (paragraph 26). The test was performed on five (5) *Wistar* strain female rats. Once the group was formed, the animals were acclimatised for 07 days after which they received a single dose of 5000 mg/kg body weight of the methanol extract of the *Piper capense* fruits by endogastric gavage. These animals were then observed with particular attention to their behaviour and any other signs that might indicate the toxic effect of the plant extract for 4 h after gavage. After 48 h, LD50 was determined from the data obtained and the animals were left for observation for an additional fourteen (14) days before being terminated for macroscopic observation of the different organs (OECD, 2008a).

### 2.5 Subchronic oral toxicity study

A total of forty (40) male and female *Wistar* strain albino rats were divided into 4 groups of 10 animals each (5 males and 5 females). Animals in group I received 7% TWEEN as the control group while those in groups II, III, and IV received the methanol extract from *P. capense* fruits, respectively, at doses 250, 500, and 1000 mg/kg body weight/day for 28 consecutive days. The animals were weighed after each treatment and the amount of feed consumed by each rat was measured daily. The study protocol was based on the recommendations of the OECD (OECD, 2008b).

### 2.6 Collection of urine, blood, serum and organs

On the last day of treatment, the animals were subjected to a fasting period of 12 h after which they were weighed and their urine collected. These animals were anaesthetized with ketamine (0.35 mL/kg) and xylazine (0.10 mL/kg) intraperitoneally, and blood was collected by cardiac puncture into two different tubes: EDTA (Ethylene diamine tetra-acetic acid) tubes for the evaluation of haematological parameters and haemolysis tubes for collecting serum which was obtained after centrifugation of blood at 3500 rpm for 10 min.

### 2.7 Determination of serum enzymes and haematological parameters

Using an automated system (QBC Autoread Plus, United Kingdom), the whole blood collected in EDTA tubes was used to determine the following haematological parameters: white blood cells (WBCs), red blood cells (RBCs), hematocrit (Hct), Platelets (Plt), Haemoglobin (Hgb), Mean corpuscular haemoglobin (MCH), Mean corpuscular haemoglobin concentration (MCHC), Mean corpuscular volume (MCV),Granulocytes (Gran), Lymphocytes (Lym), mean platelet volume (MPV), mid-cells (Mid).

The serum obtained after blood centrifugation was used for the evaluation of the following biochemical parameters: ALAT, ASAT, creatinine, urea, total protein, total cholesterol, High Density Lipoprotein (HDL) cholesterol and triglycerides using *SGM Italia kits*. The urine collected was used for the evaluation of creatinine, urea and total urinary protein using the same commercial kits. Low Density Lipoprotein (LDL) cholesterol was determined by calculation using the Friedewald equation (Friedewald et al., 1972).

### 2.8 Determination of the relative weight of the organs

Animals were weighed on an empty stomach to determine the weight gained throughout the test. The entire organs of all the animals were collected (liver, kidneys, spleen, lung and heart) and weighed to determine their relative weights according to the formula.

Relative weight of the organ (%) = (weight of the organ)/(weight of the animal) ×100.

### 2.9 Histopathological assessment

After termination, small quantities (in the left liver lobe and left kidney of each rat) of liver and kidney were preserved in 10% formalin and histopathological examination was performed by conventional methods and techniques as described by Di-Fiore (Di Fiore, 1963). After preparation of the sectioned tissues, they were filmed using a camera connected to a microscope (magnification = 400X).

### 2.10 Phytochemical screening of *Piper capense*

The main classes of secondary metabolites: alkaloids (Mayer's tests), sterols (Salkowski's test), polyphenols (ferric chloride test), tanins (gelatin test), saponins (foam test), flavonoids (aluminum chloride test), triterpenes (Liebermann-Burchard's test), and anthraquinones (Borntrager's test) were investigated following the phytochemical methods as described by Harbone (1973) and Poumale et al. (2013).

### 2.11 Statistical analysis

The analysis of variance (ANOVA) followed by the Dunnett test for multiple comparisons was performed using GraphPad Prism version 5.00; all results obtained in this study are expressed as mean ± Standard Deviation (SD) with a significant threshold of p < 0.05.

## 3 Results

### 3.1 Acute oral toxicity

During the acute toxicity study, there was no mortality recorded in animals after receiving a single dose of 5000 mg/kg body weight of *P. capense* fruit methanol extract. Also, no signs of toxicity in general appearance (reduction of locomotion, stool appearance, drowsiness, salivation, reaction to noise), were observed in animals receiving this dose after 14 days of observation. Based on the OECD principle, it can be stated that the LD50 of the methanol extract from *P. capense* fruits is greater than 5000 mg/kg body weight.

### 3.2 Subchronic oral toxicity

#### 3.2.1 Feed consumed and weight gaine

The amount of feed consumed was recorded during the test period and it appears in Table 1 that there was no significant variation in the test group compared to the control group. However, the result shows the ability of the extract to induce an increase in weight in male rats at doses 250 and 500 mg/kg body weight/day and a slight weight gain at dose 1000 mg/kg body weight/day. In female rats the extract induced a slight weight gain at 250 and 500 mg/kg body weight/day.

**Table 1:** Food consumption and weight gained after 28 days of oral treatment.

Dose (mg/kg bw)	Female				Male			
	0	250	500	1000	0	250	500	1000
Food consumed (g)	1015 ± 136.2^a^	1049 ± 81.58^a^	1027 ± 126.4^a^	916.0 ± 31.12^a^	1194 ± 88.67^a^	1140 ± 152.9^a^	1181 ± 104.3^a^	1238 ± 90.08^a^
Initial weight(g)	174.3 ± 10.21	181.7 ± 2.082	154.3 ± 7.767	206.7 ± 3.512	180.0 ± 9.165	179.7 ± 6.658	182.7 ± 9.238	183.3 ± 13.58
final weight (g)	218.3 ± 2.082	217.3 ± 1.155	197.0 ± 7.211	233.0 ± 5.196	279.0 ± 17.78	288.0 ± 20.88	287.0 ± 19.16	272.0 ± 5.568

Results are expressed as mean ± standard deviation. Values in the test groups carrying the same letter as the control group in the same-sex and in the same row are not significantly different at 5%; bw: body weight.

#### 3.2.2 Relative organ weight

Table 2 shows that there is no significant difference in relative organ weight both in males and females, and between the different treatment doses.

**Table 2:** Relative organ weights of male and female rats after 28 days of oral treatment.

Dose (mg/kg)	Female				Male			
	0	250	500	1000	0	250	500	1000
Liver (g)	3.442 ± 0.348^a^	3.420 ± 0.114^a^	3.381 ± 0.052^a^	3.529 ± 0.179^a^	3.217 ± 0.124^a^	3.190 ± 0.042^a^	3.384 ± 0.091^a^	3.512 ± 0.176^b^
Heart (g)	0.333 ± 0.017^a^	0.323 ± 0.009^a^	0.342 ± 0.017^a^	0.286 ± 0.012^b^	0.283 ± 0.008^a^	0.286 ± 0.024^a^	0.278 ± 0.005^a^	0.296 ± 0.018^a^
Lung (g)	0.848 ± 0.108^a^	0.670 ± 0.067^a^	0.734 ± 0.111^a^	0.671 ± 0.079^a^	0.616 ± 0.046^a^	0.595 ± 0.024^a^	0.662 ± 0.079^a^	0.611 ± 0.060^a^
Spleen (g)	0.405 ± 0.103^a^	0.403 ± 0.047^a^	0.441 ± 0.032^a^	0.337 ± 0.045^a^	0.343 ± 0.023^a^	0.343 ± 0.022^a^	0.271 ± 0.024^b^	0.288 ± 0.022^b^
Kidney (g)	0.650 ± 0.007^a^	0.628 ± 0.029^a^	0.666 ± 0.037^a^	0.632 ± 0.021^a^	0.604 ± 0.003^a^	0.606 ± 0.071^a^	0.621 ± 0.021^a^	0.643 ± 0.010^a^

Results are expressed as mean ± standard deviation. Values in the test groups carrying the same letter as the control group in the same-sex and in the same row are not significantly different at 5%.

### 3.3 Histopathology of the liver and kidneys

**Figure 1:**
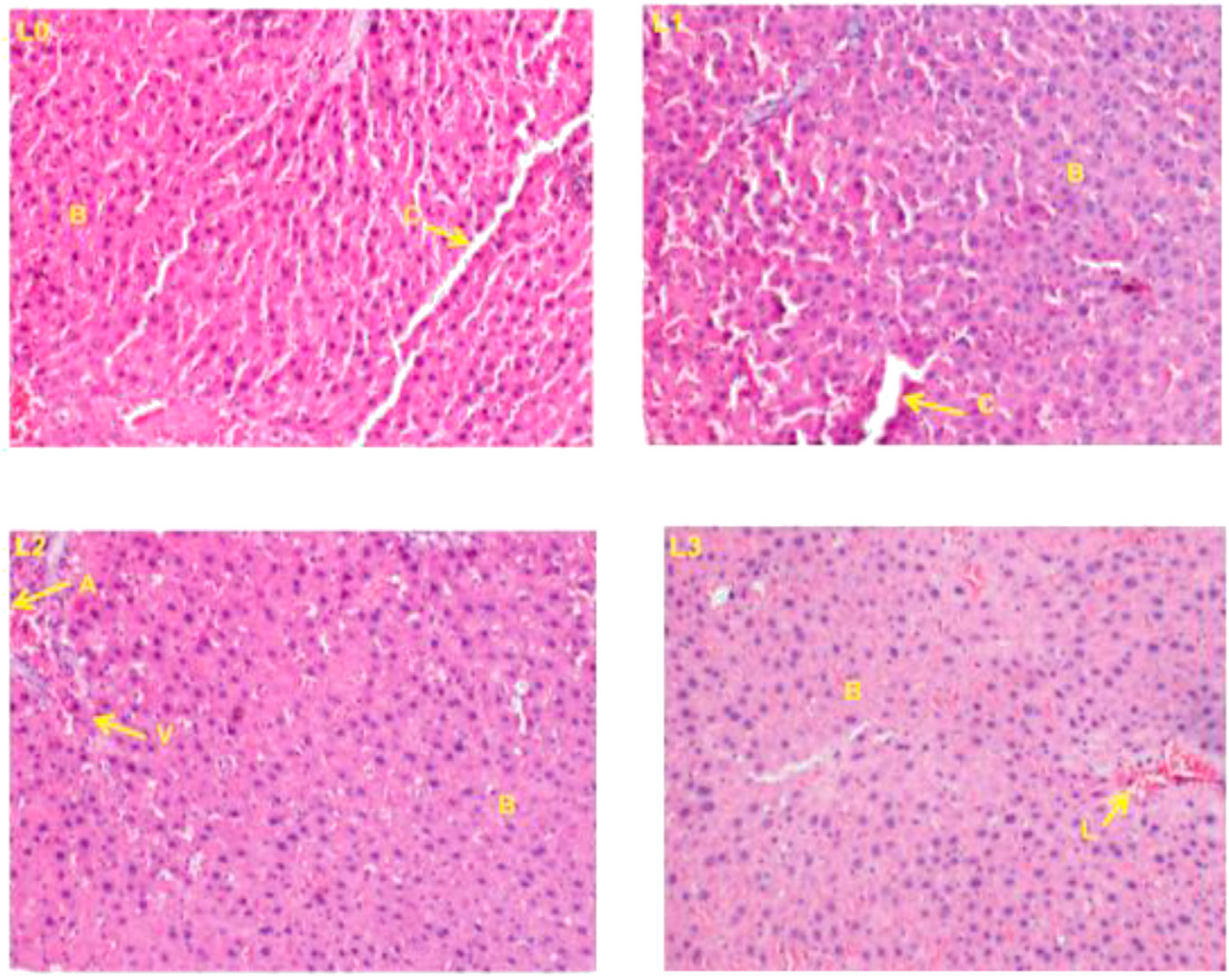
Histopathological changes in liver of rats after 30 days of treatment in males rats (400X). (L0) control; (L1) wistar strain rats treated with 250 mg/kg fruits extracts of *P. capense*; (L2) wistar strain rats treated with 500 mg/kg fruits extracts of *P. capense;* (L3) wistar strain rats treated with 1000 mg/kg fruits extracts of *P. capense s,* A: Branch of the Hepatic portal vein, B: hepatocytes, V: centro lobular vein, C: leucocytes filtration, L: Cell lysis.

**Figure 2:**
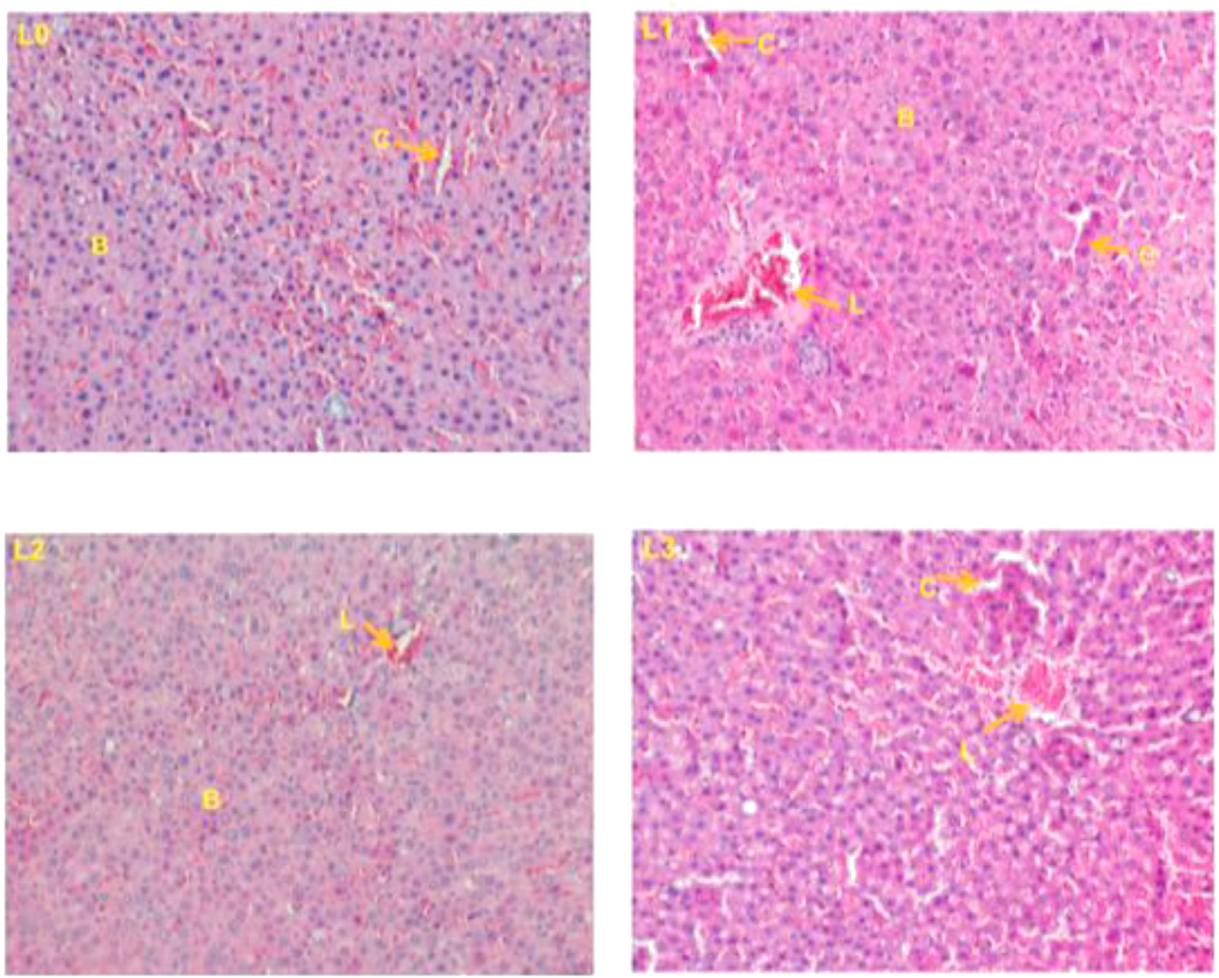
Histopathological changes in liver of rats after 30 days of treatment in female rats (400X). (L0) control; (L1) wistar strain rats treated with 250 mg/kg fruits extracts of *P. capense*; (L2) wistar strain rats treated with 500 mg/kg fruits extracts of *P. capense;* (L3) wistar strain rats treated with 1000 mg/kg fruits extracts of *P. capense s,* A: Branch of the Hepatic portal vein, B: hepatocytes, V: centro lobular vein, C: leucocytes filtration, L: Cell lysis.

**Figure 3:**
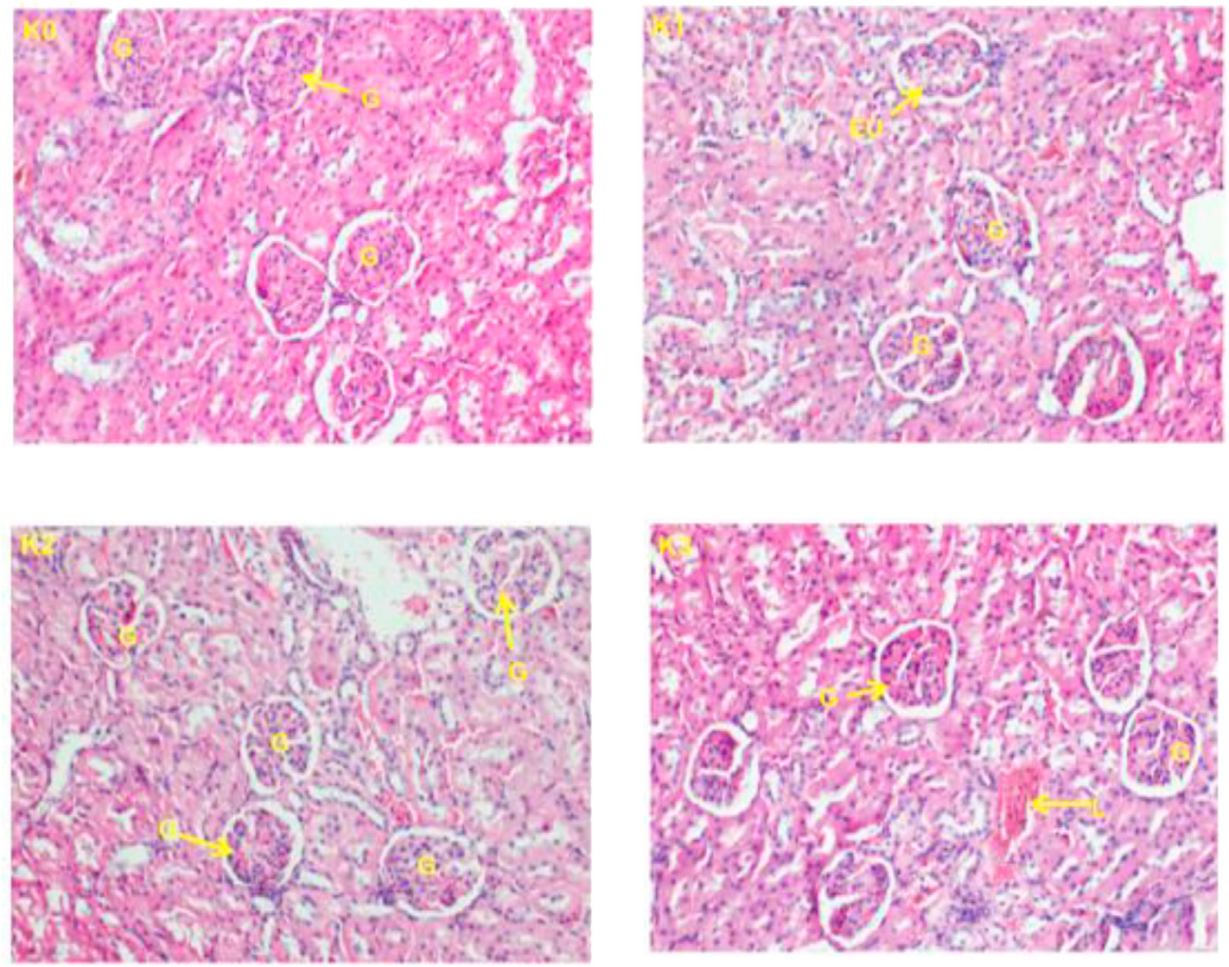
Histopathological changes in kidney of rats after 28 days of treatment in males rats (400X). (K0) control; (K1) wistar strain rats treated with 250 mg/kg fruits extracts of *P. capense*; (K2) wistar strain rats treated with 500 mg/kg fruits extracts of *P. capense;* (K3) wistar strain rats treated with 1000 mg/kg fruits extracts of *P. capense s,* L: Cell lysis, G: glomerulus, EU: urinary tract, D: distal convoluted tubule, P: proximal convoluted tubule.

**Figure 4:**
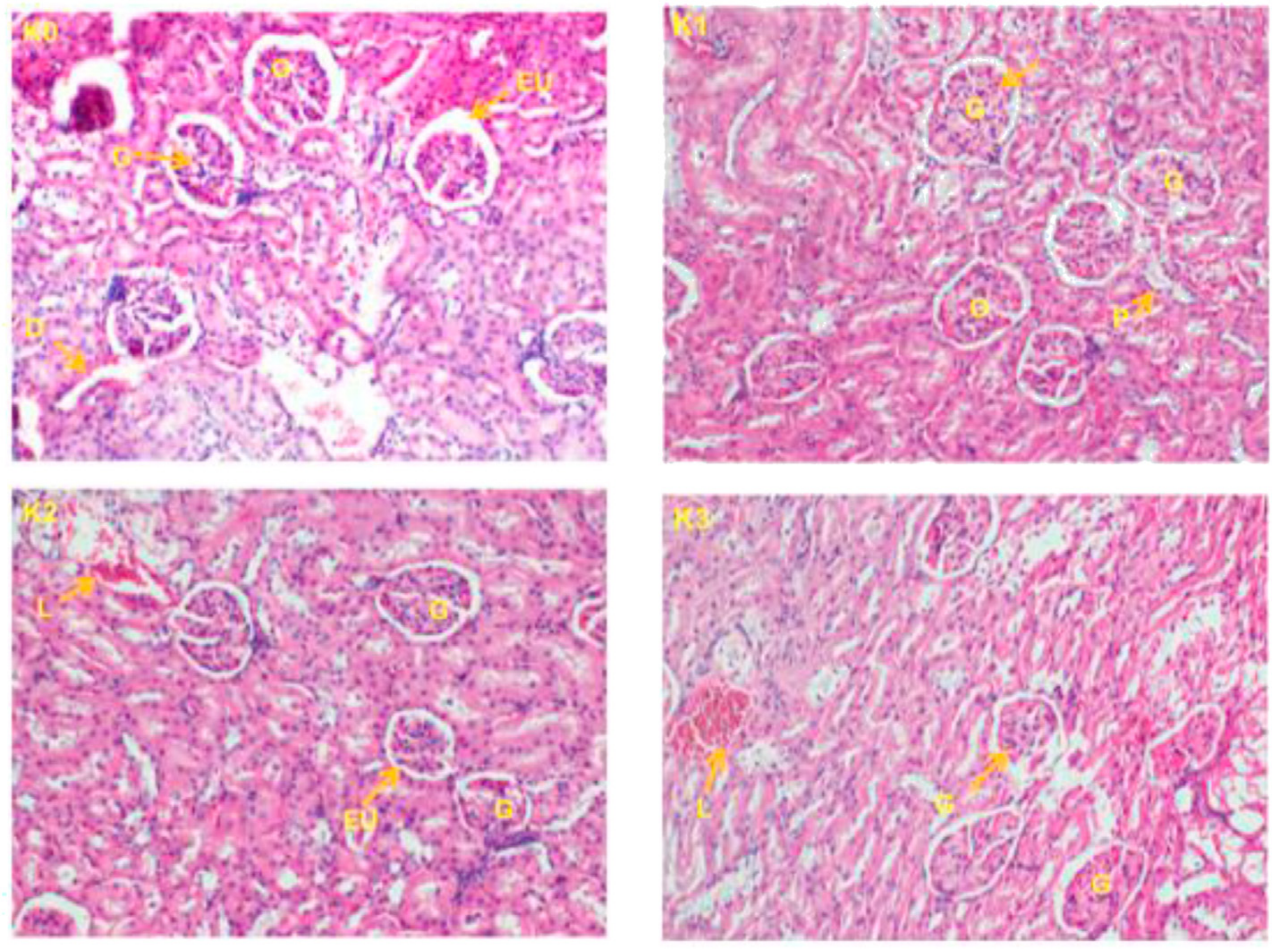
Histopathological changes in kidneyof rats after 30 days of treatment in female rats (400X). (K0) control; (K1) wistar strain rats treated with 250 mg/kg fruits extracts of *P. capense*; (K2) wistar strain rats treated with 500 mg/kg fruits extracts of *P. capense;* (K3) wistar strain rats treated with 1000 mg/kg fruits extracts of *P. capense s,* L: Cell lysis, G: glomerulus, EU: urinary tract, D: distal convoluted tubule, P: proximal convoluted tubule.

Histopathological examination was performed on the kidneys and liver of the males and females to detect morphological damages on them. The histological sections performed are presented in Figures 1 and 2 for the liver in male and female rats respectively, while Figures 3 and 4 represent the histological sections of kidneys of the male and female rats respectively. Observation of these cuts did not reveal any abnormalities on the kidneys at doses 0 and 250 mg/kg body weight/day in both males and females, but at doses 500 and 1000 mg/kg body weight/day in both sexes cell lysis was observed. Concomitantly, the observation of the liver reveals some slight alterations, namely leukocyte infiltration and cell lysis at all doses and in both sexes.

### 3.4 Haematological parameters

The results of haematological parameters (Table 3) show a significant decrease of WBCs count in females at dose 1000 mg/kg body weight/day compared to the control group, while the amount of lymphocytes increased significantly at the same dose. In males, there was a significant increase of WBCs, Gran, MCV, MCH and MCHC amounts at 500 and 1000 mg/kg body weight/day, but a significant decrease in Lym amount at 250, 500 and 1000 mg/kg body weight/day. We also observed a significant decrease of RBCs counts and Hct at 500 and 1000 mg/kg body weight/day compared to the control group.

**Table 3:** Heamatological parameters in male and female rats after 28 days of oral treatment.

Dose (mg/kg)	Female				Male			
	0	250	500	1000	0	250	500	1000
WBCs (10^3^/μL)	18.60 ± 0.458^a^	21.83 ± 3.235^a^	18.23 ± 2.084^a^	12.47 ± 2.631^b^	18.30 ± 0.900^a^	19.60 ± 2.193^a^	21.77 ± 0.650^b^	25.50 ± 1.100^c^
Lym (%)	74.70 ± 1.353^a^	73.73 ± 2.650^a^	78.33 ± 2.868^a^	82.93 ± 5.479^b^	77.85 ± 0.250^a^	71.70 ± 3.559^b^	45.10 ± 0.984^c^	49.40 ± 3.600^c^
Mid (%)	11.80 ± 2.095^a^	12.33 ± 0.896^a^	12.43 ± 1.419^a^	12.73 ± 1.258^a^	12.75 ± 0.550^a^	12.57 ± 0.814^a^	11.93 ± 1.704^a^	12.20 ± 1.500^a^
Gran (%)	15.83 ± 1.815^a^	13.30 ± 3.20^a^	13.23 ± 4.302^a^	10.75 ± 0.950^a^	17.90 ± 1.200^a^	18.07 ± 4.102^a^	42.53 ± 1.650^b^	38.40 ± 5.100^b^
Plt (10^3^/μL)	581.0 ± 0.00^a^	500.5 ± 55.50^a^	562.3 ± 37.98^a^	546.0 ± 20.0^a^	737.0 ± 94.00^a^	681.0 ± 73.74^a^	864.3 ± 60.17^a^	762.0 ± 77.0^a^
VMP (fL)	10.47 ± 1.850^a^	10.07 ± 2.294^a^	11.63 ± 0.981^a^	10.47 ± 1.861^a^	11.30 ± 1.000^a^	9.000 ± 0.700^a^	11.47 ± 1.301^a^	11.10 ± 0.700^a^
RBCs (10^6^/μL)	6.960 ± 1.099^a^	7.397 ± 0.347^a^	5.797 ± 1.255^a^	7.090 ± 0.661^a^	6.405 ± 0.145^a^	6.597 ± 0.251^a^	1.020 ± 0.396^b^	0.805 ± 0.075^b^
Hgb (g/dL)	16.30 ± 0.360^a^	16.97 ± 1.570^a^	14.33 ± 2.303^a^	16.03 ± 1.401^a^	17.00 ± 1.200^a^	17.20 ± 1.044^a^	15.77 ± 0.808^a^	16.70 ± 0.900^a^
Hct %	41.37 ± 5.299^a^	41.17 ± 4.105^a^	38.67 ± 2.003^a^	41.13 ± 3.803^a^	38.35 ± 0.850^a^	38.77 ± 4.500^a^	11.63 ± 1.419^b^	7.400 ± 0.700^b^
MCV (fL)	59.80 ± 3.051^a^	55.63 ± 3.625^a^	61.07 ± 0.230^a^	58.17 ± 3.609^a^	63.38 ± 3.852^a^	60.03 ± 7.995^a^	88.13 ± 1.877^b^	92.75 ± 0.150^b^
MCH (pg)	23.77 ± 3.667^a^	22.87 ± 1.201^a^	24.97 ± 2.413^a^	22.57 ± 0.750^a^	23.25 ± 1.250^a^	23.50 ± 1.00^a^	130.5 ± 17.20^b^	208.2 ± 8.200^c^
MCHC (g/dL)	39.73 ± 4.524^a^	41.27 ± 3.647^a^	40.97 ± 3.953^a^	38.97 ± 1.155^a^	50.15 ± 3.950^a^	44.57 ± 3.884^a^	146.9 ± 18.40^b^	226.5 ± 9.300^c^

Results are expressed as mean ± standard deviation. Values in the test groups carrying the same letter as the control group in the same-sex and in the same row are not significantly different at 5%; WBCs: White Blood Cells, RBCs: Red blood cells, Hct: Hematocrit, Plt: Platelets, Hgb: Haemoglobin, MCH: Mean corpuscular haemoglobin, MCHC: Mean corpuscular haemoglobin concentration, MCV: Mean corpuscular volume, Gran: Granulocytes, Lym: Lymphocytes, MPV: mean platelet volume, Mid: mid-cells.

### 3.5 Serum and urinary biochemical parameters

The results of the serum and urinary biochemical parameters of rats treated with methanol extract from *P. capense* fruits are presented in Tables 4 and 5. The extract did not affect urinary urea levels in either sexes. Serum urea levels were not modified in female rats, while an increase in serum urea was observed in male rats in all test groups (250, 500 and 1000 mg/kg body weight/day). Total serum and urinary proteins were not modified in males, while in females, there was a significant increase at 1000 mg/kg body weight/day and a significant decrease at 250, 500 and 1000 mg/kg body weight/day respectively. Serum creatinine levels were not modified in either sexes, as it was the case of urinary creatinine levels in female rats; while the male rats showed a significant and progressive decrease of urinary creatinine at doses 250, 500 and 1000 mg/kg body weight/day. The amount of ALAT was not modified by the extract in either sexes, while that of ASAT was not equally modified in males as opposed to females. We observed a significant decrease between the control dose and two test groups (the groups of animals which received 250 and 500 mg/kg body weight/day). The lipid profile of the rats obtained through the evaluation of total cholesterol, triglycerides, LDL cholesterol and HDL cholesterol showed a progressive and significant decrease in the quantity of triglyceride at different doses in both sexes; an increase in the amount of LDL cholesterol in female rats but with a decrease of the same parameters in male rats at all doses was equally observed. No changes were observed in the concentration of total cholesterol and HDL cholesterol in either sexes.

**Table 4:** Serum and urinary biochemical parameters in male and female after 28 days of oral treatment.

Sex	Female				Male			
Dose (mg/kg)	0	250	500	1000	0	250	500	1000
**Serum**								
Creatinine (mg/dL)	0.650 ± 0.027^a^	0.682 ± 0.072^a^	0.619 ± 0.074^a^	0.523 ± 0.047^a^	0.666 ± 0.095^a^	0.587 ± 0.068^a^	0.634 ± 0.031^a^	0.682 ± 0.069^a^
Proteins (g/dL)	4.714 ± 0.391^a^	4.102 ± 0.362^a^	4.456 ± 0.423^a^	6.374 ± 0.283^b^	5.190 ± 0.902^a^	4.510 ± 0.479^a^	5.014 ± 0.047^a^	4.959 ± 0.409^a^
Urea (mg/dL)	51.79 ± 6.405^a^	55.90 ± 0.444^a^	57.21 ± 1.167^a^	52.88 ± 1.574^a^	34.87 ± 1.601^a^	41.10 ± 1.157^b^	45.90 ± 3.203^b^	45.90 ± 4.945^b^
ALAT (UI/L)	22.87 ± 0.870^a^	24.37 ± 1.997^a^	23.17 ± 0.275^a^	24.43 ± 0.881^a^	22.80 ± 0.984^a^	21.24 ± 2.510^a^	24.60 ± 2.790^a^	21.79 ± 2.846^a^
ASAT (UI/L)	42.62 ± 0.556^a^	35.26 ± 1.320^b^	36.96 ± 2.368^b^	42.43 ± 1.619^a^	55.77 ± 3.787^a^	57.46 ± 0.635^a^	53.69 ± 0.039^a^	54.68 ± 3.495^a^
**Urine**								
creatinine (mg/dL)	139.8 ± 27.28^a^	116.9 ± 39.66^a^	108.5 ± 12.59^a^	93.69 ± 16.15^a^	233.2 ± 27.01^a^	173.0 ± 13.79^b^	138.4 ± 17.32^b^	86.13 ± 19.41^b^
proteins (g/dL)	1.680 ± 0.163^a^	0.510 ± 0.102^b^	0.596 ± 0.051^b^	1.007 ± 0.129^b^	0.707 ± 0.112^a^	0.591 ± 0.034^a^	1.361 ± 0.239^b^	0.583 ± 0.062^a^
Urea (mg/dL)	356.4 ± 48.09^a^	423.1 ± 68.05^a^	276.9 ± 55.87^a^	323.1 ± 37.88^a^	751.5 ± 11.84^a^	687.0 ± 13.48^a^	546.2 ± 110.1^a^	674.4 ± 125.0^a^

Results are expressed as mean ± standard deviation. Values in the test groups carrying the same letter as the control group in the same-sex and in the same row are not significantly different at 5%; ALAT: alanine aminotransferase; ASAT: aspartate aminotransferase.

**Table 5:** Lipid profile of male and female rats after 28 days of oral treatment.

Dose (mg/kg)	Female				Male			
	0	250	500	1000	0	250	500	1000
TC (mg/dL)	104.6 ± 0.652^a^	115.2 ± 5.882^a^	114.1 ± 5.745^a^	107.0 ± 0.500^a^	108.4 ± 5.092^a^	107.8 ± 7.402^a^	105.5 ± 3.286^a^	109.1 ± 1.146^a^
HDL-C (mg/dL)	55.82 ± 4.030^a^	57.81 ± 2.762^a^	55.72 ± 3.257^a^	54.44 ± 0.629^a^	52.28 ± 1.528^b^	52.93 ± 3.339^b^	52.84 ± 1.194^b^	63.15 ± 1.279^a^
Triglyceride (mg/dL)	50.26 ± 1.789^a^	34.10 ± 1.398^c^	36.50 ± 0.318^b^	37.67 ± 0.892^b^	67.06 ± 4.143^a^	57.62 ± 1.667^b^	53.57 ± 3.223^b^	54.08 ± 0.119^b^
LDL-C (mg/dL)	42.34 ± 0.917^b^	46.11 ± 2.420^b^	44.83 ± 3.233^b^	49.39 ± 3.117^a^	48.13 ± 5.537^a^	41.30 ± 3.500^a^	38.69 ± 2.057^b^	37.18 ± 3.673^b^

Results are expressed as mean ± standard deviation. Values in the test groups carrying the same letter as the control group in the same-sex and in the same row are not significantly different at 5%; TC: Total cholesterol; HDL-C: HDL-cholesterol; LDL-C: LDL-cholesterol.

### 3.6 Qualitative phytochemical composition of *Piper capense* extract

The results of the phytochemical screening reported in Table 6 revealed the presence of alkaloids, polyphenols, saponins, flavonoids, tannins and sterols in the plant extract while secondary metabolites such as anthraquinones, and triterpenes were absent in the plant extract.

**Table 6:** Phytochemical composition of *Piper capense* extract.

Chemical classes	*Piper capense* extract
Extractive yield (%)	12.80
Alkaloids	+
Anthraquinones	-
Flavonoids	+
Polyphenols	+
Saponins	+
Tannins	+
Sterols	+
Triterpenes	-

(+): Present; (-): Absent; yield is obtained by the ratio of the mass of the extract to the methanol obtained to the mass of the plant powder.

## 4 Discussion

Today we are increasingly witnessing not only a resurgence of diseases that were once controlled by the scientific community, but also the emergence of new diseases causing a significant number of deaths worldwide. In the search for ways and means to deal with these diseases, scientists are increasingly turning to natural sources (plants) as this is considered a field that is diversifying due to secondary metabolites and very little has been explored so far. Large groups of secondary metabolites are the target of scientists because they are known for their diverse biological activities. However, these same compounds with significant toxic effects well established in literature (Bello et al., 2016) may be harmful to human organisms as they are considered to be the main beneficiaries. The search of plants with the best bioactive compounds including the data on the study of their toxicity is therefore an absolute necessity. That is why this work with the aim of evaluating the acute and subchronic oral toxicities of the methanol extract from *P. capense* fruits used in Cameroon as an edible plant was carried out. Several studies have demonstrated its biological activity, but none of the studies has evaluated its toxicity, hence the importance of this work.

Based on the limit dose of 5000 mg/kg, the methanol extract from the fruit of *P. capense* was considered non-toxic (Delongeas et al., 1983). No morbidity or mortality nor toxic signs were detected over the 14 days observation period.

The result of acute toxicity permitted us to conduct a subchronic oral toxicity study of the *P. capense* fruits methanol extract.

A 28-day oral administration was performed with *P. capense* methanol extract on *wistar* strain albino rats, at the end of which, a number of parameters were evaluated to assess its toxic effects. No deaths were recorded during the test period. The amount of food consumed during the 28 days of treatment was measured and a slight variation but not significant was observed in both sexes.

Several classes of secondary metabolites in plants are known to have cytotoxic and antitumour activities, including alkaloids, polyphenols, saponins, flavonoids, tannins, and sterols (Kinghorn, 2000; Birudu and Naik, 2014). The presence of alkaloids, polyphenols, saponins, flavonoids, tannins and sterols in the methanol extract of *Piper capense* may justify the various biological activities of this plant. The phytochemical screening results obtained in this study corroborate those of Fankam et al. (2011). However, contrary to Fankam and co-workers (2011), the flavonoids were detected in the methanol extract of *Piper capense* tested herein.

The performance of an experiment in which rats are exposed to a toxic product results in damage, either by an increase or decrease in the relative weight of the organs of metabolism such as liver, kidneys, spleen, heart, and lung (Busari et al., 2015). In this study, there was virtually no significant difference in the relative weight of liver, kidneys, spleen, heart, and lung organs between the test groups and those of the control group. So, based on the relative weight of the organs we can conclude that the methanol extract from *P. capense* fruits has low toxicity at high doses.

Anemia can occur in animals following administration of a product resulting from lysis of blood cells or inhibition of their synthesis by bioactive components contained in this product (Onyeyilli et al., 1998). In this study, after administration of the extract from *P. capense* at different doses in rats, there was no significant difference between the haematological parameters of the test groups compared to those of the control group in female rats. However, in male rats some differences were observed, including an increase in WBCs and Granulocyte levels at doses 500 and 1000 mg/kg body weight/day. This could be attributed to the boosting capacity of the immune system of the test animals by the bioactive molecules in the extract (Idoh et al., 2016). The significant decrease in RBCs and hematocrit levels in male rats compared to control group could indicate a renal damage caused by methanol extract from *P. capense* fruits at doses 500 and 1000 mg/kg body weight/day. A possible explanation would be the insufficient erythropoietin production impeding the synthesis of RBCs by the bone marrow due to the adverse effect of the biologically active molecules contained in the extract at these doses (Cloutier et al., 2014).

The primary function of the kidneys is to eliminate toxic waste produced by the normal functioning of the body and transported by the blood. Therefore, the evaluation of its excretory products accompanied by histological studies give enough information on the proper functioning of the kidneys. Also, an abnormal increase in creatinine or serum urea level would be the consequence of a renal damage caused by *Piper capense* extract (Schaffer and Menche, 2004; Palm and Lundblad, 2005). In this study, no significant difference was observed in serum creatinine levels between the control and test groups in either sexes. The amount of urea did not differ in female rats (no significant difference) between the control and the test groups, while there was a significant increase at different treatment doses compared to the control group in male rats. We also observed a significant decrease in urinary protein levels in female rats and in urinary creatinine levels in male rats. These results suggest a possible renal alteration of male rats following the consumption of *P. capense* fruits extract.

A significant variation in serum ALAT, ASAT and protein levels following long-term exposure of a product indicates its harmful or damaging effect on the liver, either by altering the membrane permeability of liver cells or by inducing necrosis by this product (Giannini et al., 2005; Hyder et al., 2013). In this study, the oral administration of the methanol extract of *P. capense* fruits at different doses resulted in a non-significant decrease in ASAT. Also, the amount of ALAT and total serum proteins were not affected in the test groups compared to the control group. This could indicate the hepatoprotective effect of the extract because Peirs (2005) correlated the hepatoprotective activity of a product with its ability to induce the decrease in one or more transaminases.

The lipid profile is a set of parameters among which LDL cholesterol, total cholesterol and triglycerides are those mainly involved in the risk factors of cardiovascular diseases (Schaffer and Menche, 2004). The decrease in LDL cholesterol and triglyceride levels in male rats as well as the maintenance of HDL cholesterol levels allow us to conclude that methanol extract from *P. capense* fruits at different treatment doses would not cause any risk of cardiovascular diseases occurrence.

The main organs subjected to damage following exposure of an organism to a product are the liver (as the main organ of metabolism) and the kidneys (as the main organ of elimination). The histopathological examinations performed on these two organs in this study showed some slight alterations both on the liver and kidneys at doses 500 and 1000 mg/kg body weight/day but there were no signs of toxicity following a long exposure (28 days) of the extract to methanol from *P. capense* fruit because no damage has been observed on these two organs in either sexes.

## 5 Conclusions

These data suggest that methanol extract from *Piper capense* fruits is non-toxic at doses lower than 1000 mg/kg body weight/day but could nevertheless be toxic at doses above 1000 mg/kg body weight/day; therefore, we strongly discourage its consumption at higher doses for therapeutic purposes. Finally, this edible plant is a potential pharmaceutical agent that deserves further in-depth toxicity studies. One of the limitations of this study is the characterization of the phytochemical constituents, and mostly the bioactive as well as potentially toxic constituents of the tested methanol extract. However, this work is ongoing and constitutes the aim of our further investigations.

## Declarations

### Author contribution statement

V. Kuete: Conceived and designed the experiments; Contributed reagents, materials, analysis tools or data; Wrote the paper.

B.E.N. Wamba: Performed the experiments; Analyzed and interpreted the data; Contributed reagents, materials, analysis tools or data; Wrote the paper.

A.T. Mbaveng: Conceived and designed the experiments; Contributed reagents, materials, analysis tools or data.

G.M. Tazoho: Analyzed and interpreted the data.

### Funding statement

This research did not receive any specific grant from funding agencies in the public, commercial, or not-for-profit sectors.

### Declaration of interests statement

The authors declare no conflict of interest.

### Additional information

No additional information is available for this paper.
